# Increased Levels of Serum Neuregulin 1 Associated with Cognitive Impairment in Vascular Dementia

**DOI:** 10.1155/2020/6683747

**Published:** 2020-11-12

**Authors:** Xiaoling Wang, Fengyu Zhang, Weibin Ma, Depeng Feng, Jingjing Zhang, Jialei Xu

**Affiliations:** Department of Neurology, Liaocheng People's Hospital, Liaocheng, Shandong, China 252000

## Abstract

**Objective:**

Neuregulin 1 (NRG 1) is a member of the epidermal growth factor (EGF) family and is believed to play an important role in neuroplasticity. However, the relationship between NRG 1 and vascular dementia (VaD) is poorly understood. The purpose of this study is to explore the correlation between neuregulin 1 and VaD. *Patients and Methods*. From October 2018 to September 2020, 93 VaD patients and 79 control populations who attended Liaocheng People's Hospital were included in the study. Baseline characteristics including age, gender, years of education, HDL, LDL, FBG, SBP, and DBP are collected. At the same time, peripheral blood was collected, and the concentration of serum NRG 1 was detected by enzyme-linked immunosorbent assay (ELISA). All research subjects received professional cognitive function assessment.

**Results:**

A total of 93 VaD patients and 79 controls were enrolled. There was no significant difference in age, gender, years of education, HDL, LDL, FBG, SBP, and DBP between the two groups (*p* > 0.05). However, compared with the control group, VaD patients have lower MoCA and higher serum NRG 1 levels, and the difference is statistically significant (*p* < 0.001). The correlation analysis of MoCA and baseline characteristics showed that the MoCA score in VaD was significantly negatively correlated with serum NRG 1 (*r* = −0.374, *p* = 0.036). The results of multivariate regression showed that the MoCA score of VaD patients was only associated with NRG 1 (*β* = 0.258, *p* = 0.012).

**Conclusions:**

The concentration of serum NRG 1 in VaD patients is significantly increased, which may be an independent risk factor for cognitive impairment in VaD patients.

## 1. Introduction

Vascular dementia (VaD) is one of the main causes of dementia syndrome and is defined as the impairment of cognitive function due to vascular disorders caused by impaired cerebral blood flow [1, 2]. There are currently 35.6 million dementia patients worldwide, and VaD is the subtype of dementia second only to Alzheimer's disease (AD) [3–5]. With the increase of life expectancy, neurovascular disorders are threatening human physical and mental health, and VaD is a public health issue that needs to be solved urgently [6]. There is currently no cure for VaD [7]. Therefore, finding low-invasive and high-efficiency biomarkers is a hot spot for clinical and scientific researchers.

Neuregulin 1 (NRG 1) is a trophic factor that contains an epidermal growth factor- (EGF-) like domain [8]. The gene encoding NRG 1 is 2.4 Mbp (chromosome 8) in mice and 2.6 Mbp in humans (chromosome 8) and rats (chromosome 16). NRG 1 encoded by the NRG 1 gene has at least 31 subtypes and 6 types of NRG 1 (I to VI), due to alternative splicing, and has been identified as a member of the active epithelial growth factor (EGF) family [9]. Most NRG 1 subtypes are synthesized as pro-NRG-1, which binds to the cell membrane and is contained in the extracellular EGF domain, which can be cleaved by three transmembrane NRG 1 proteolytic enzymes (ADAM17, BACE, and ADAM19) and then released into extracellular fluid as soluble NRG 1 [10]. These types or isomers perform a wide range of functions in the body and are considered to be key factors in neurodevelopment [11, 12]. The specific binding of NRG 1 to its receptor ErbB can activate a series of different pathophysiological processes, including maintaining myelin sheath stability, promoting neurite growth, and regulating cell proliferation, differentiation, and apoptosis [13].

More and more studies suggest that NRG 1 is involved in many neurological diseases, but its underlying mechanism is still unclear. The purpose of our current research is to (1) explore the relationship between NRG 1 and VaD and (2) clarify whether NRG 1 can be used as a biomarker of VaD. These issues have not been reported in previous studies. The determination of the correlation between NRG 1 and vascular cognitive impairment will provide new possibilities for the treatment of VaD.

## 2. Patients and Methods

### 2.1. Study Population

93 VaD patients and 79 normal controls who were treated in Liaocheng People's Hospital from October 2018 to September 2020 were used as the study population. The diagnostic criteria of VaD refer to the National Institute for Neurological Disorders and Stroke (NINDS-AIREN) and International Classification of Diseases, 11th revision (ICD-11) [14, 15]. All VaD diagnoses are made by an experienced neurologist. The exclusion criteria are as follows: (1) mixed dementia; (2) active neuroimmune disease, rheumatic system disease, or acute infection; (3) history of acute stroke and tumor; (4) mental diseases such as schizophrenia; and (5) the patient or family members do not cooperate with the examination. This study was authorized by the Ethics Committee of Liaocheng People's Hospital. The patients and their families knew the contents of the study and signed an informed consent form. Our research complies with the Declaration of Helsinki.

### 2.2. Baseline Characteristics

When the study population visited a doctor, their baseline data were collected and recorded in detail. The main baseline information includes the following aspects: age, gender, years of education, high-density lipoprotein (HDL), low-density lipoprotein (LDL), fasting blood glucose (FBG), systolic blood pressure (SBP), and diastolic blood pressure (DBP). Demographic information such as age, gender, and years of education is collected mainly by inquiries, and the subjects of inquiries are patients or guardians who are familiar with the patient's situation. The acquisition of clinical biochemical indicators such as HDL, LDL, FBG, SBP, and DBP adopts standardized laboratory testing methods.

### 2.3. Serum NRG 1 Test

The detection of serum NRG 1 uses a commercial ELISA reagent (NRG 1, Abcam, Cambridge, MA). All study populations were collected 5 ml of peripheral blood by nurses within 24 hours after visiting a doctor. The peripheral blood was allowed to stand at room temperature for half an hour and then centrifuged at 3000 rpm for 12 minutes in a cryogenic centrifuge. The serum was separated and kept in a refrigerator at -80°C for later use. The operation process of ELISA refers to previous reports and product instructions [16, 17].

### 2.4. Cognitive Function Test

The Montreal Cognitive Assessment (MoCA) is a widely used screening assessment for detecting cognitive impairment established by Ziad Nasreddine in Montreal, Quebec, in 1996. Its reliability and validity have been confirmed and adopted by many countries. The MoCA test takes about 10 minutes and is a test with a total score of 30 points. MoCA can measure the following cognitive domains: short-term memory recall task, visuospatial abilities, multiple aspects of executive functions, attention, concentration, and working memory, language, abstract reasoning, and orientation. A MoCA score of 26 and above is considered normal [18, 19]. The MoCA evaluation is carried out by an experienced neurologist who does not know the evaluation subject's baseline date.

### 2.5. Statistical Analysis

All data statistics in this study used SPSS 22.0 software (SPSS Inc., IL, USA). All data are expressed as *n* or mean ± standarddeviation (SD). This study is aimed at detecting significant changes between VaD patients and controls. The Shapiro-Wilk test is used to assess the normality of the data distribution. The statistics of categorical variables used *χ* test, and the statistics of continuous variables used Student's test. Spearman's rank correlation is used to assess the relationship of variables. A multiple regression model was established to screen independent risk factors for cognitive impairment in VaD patients. A two-tailed *p* value of 0.05 was considered statistically significant.

## 3. Results

### 3.1. Baseline Characteristics

Baseline data of 93 VaD patients and 79 control populations were collected, and the results are shown in Table 1. The results of statistical analysis showed that regarding baseline data (age, gender, years of education, HDL, LDL, FBG, SBP, and DBP), there was no significant difference between the control group and the VaD group (*p* > 0.05). The MoCA scores of the control group and the VaD group were (27.8 ± 1.1) and (20.9 ± 1.8) points, respectively, and there were significant differences between the groups (*p* < 0.001). The serum NRG 1 concentrations of the control group and the VaD group were (264.8 ± 14.7) pg/ml and (396.8 ± 19.9) pg/ml, respectively, and there were also significant differences between the groups (*p* < 0.001).

### 3.2. Spearman's Correlation Analysis

In order to explore the related factors of cognitive impairment in VaD patients, we performed Spearman's correlation analysis of MoCA score and baseline data. The results of Spearman's correlation analysis are shown in Table 2. From the results of statistical analysis, we can see that the MoCA score of VaD patients is significantly negatively correlated with the serum NRG 1 concentration (*r* = −0.374, *p* = 0.036). However, there is no obvious correlation between MoCA score and age, gender, years of education, HDL, LDL, FBG, SBP, and DBP (*p* > 0.05).

### 3.3. Multivariate Regression Analysis

To verify the independent risk factors for cognitive impairment in VaD patients, multivariate regression analysis was used. The statistical results of multivariate regression analysis are shown in Table 3. The results of regression analysis showed that after adjusting for age, gender, years of education, HDL, LDL, FBG, SBP, and DBP and other risk factors, serum NRG 1 concentration is still an independent predictor of cognitive impairment in VaD patients (*β* = 0.258, *p* = 0.012).

## 4. Discussion

The main purpose of the current study is to study the correlation between the cognitive function and serum NRG 1 levels in normal control groups and VaD patients. The results of the study showed that the serum NRG 1 level of VaD patients was significantly higher than that of the control population, while the MoCA score was significantly lower. Our further research also found that the MoCA score in VaD patients was significantly negatively correlated with serum NRG 1 concentration, and there was no interference with other clinical baseline data such as age, gender, years of education, HDL, LDL, FBG, SBP, and DBP. As far as we know, this is the first report to study serum NRG 1 as a biomarker for predicting cognitive impairment in VaD patients.

Neuregulin 1 is involved in the process of a series of neurological diseases [20]. A Shantou University study showed that spinal cord injury can induce pathophysiological changes in brain functional areas by regulating NRG 1 signals, thereby aggravating brain injury, indicating that exogenous NRG 1 may be a potential method for the treatment of spinal cord injury [21]. The same research team found that treatment of primary mouse neurons with recombinant NRG 1 can reduce oxidative stress caused by H_2_O_2_ and inflammation caused by LPS [22]. Simmons et al. further confirmed the anti-inflammatory effect of NRG 1 in a cerebral ischemia model [23]. Interestingly, Spanish scholars also recently discovered the neuroprotective effect of NRG 1 in stroke models [24]. However, the signal pathway and neural network through which NRG 1 exerts neuroprotective effects are still unclear.

NRG 1 and ErbB4 are expressed in multiple regions of the adult brain, and NRG 1/ErbB4 plays an important role in neurodevelopment, neurotransmission, and synaptic plasticity. ErbB4 can colocalize and interact with the postsynaptic scaffold protein PSD95, which is essential for glutamatergic transmission and short-term plasticity. The regulation of GABA and ACh by NRG 1 is one of the important mechanisms for maintaining long-term plasticity [25]. A study showed that downregulation of NRG 1/ErbB4 signal is an important means to improve hippocampal learning and memory function [26]. Another study showed that the interaction of NRG 1 and ErbB4 can reduce the apoptosis of hippocampal neurons [27]. NRG 1 is involved in a number of pathophysiological processes, and the mechanism of its neuroprotective effect is far from elucidated.

Interestingly, the correlation between NRG 1 and AD has been widely reported in recent years. The study of Jiang et al. pointed out that NRG 1 has a preventive effect on AD, which provides a new perspective for the treatment of AD [28]. Xu team's research found that NRG 1 can improve cognitive impairment in AD [29]. The correlation between NRG 1 and AD has been reported in a number of studies [30–32]. These studies involve animal experiments and clinical studies, suggesting that NRG 1 can be used as a biomarker of AD, and exogenous NRG 1 can be used as a potential treatment for AD.

Our research has some limitations: first, our research is small sample research, and the correlation between NRG 1 and VaD still needs to be further confirmed; second, we have not obtained pathological samples of brain tissue, and the expression level of NRG 1 in the tissue is unknown; finally, we do not have longitudinal follow-up results for VaD patients. However, our study reported the correlation between NRG 1 and VaD for the first time, providing a new perspective on the prevention and treatment of VaD.

## 5. Conclusions

In summary, our study found that the serum NRG 1 level of VaD patients was significantly increased. We report for the first time that serum NRG 1 levels are involved in cognitive impairment in VaD patients. We hope that the relationship between the two will be further confirmed in the future. It is of great social significance to clarify the cognitive protective effect of NRG 1 on VaD.

## Figures and Tables

**Table 1:** Baseline characteristics of patients.

	Controls (*n* = 79)	VaD (*n* = 93)	*p*
Age (years)	63.4 ± 3.7	63.2 ± 3.9	0.732
Male (*n*)	48	59	0.718
Education (years)	9.2 ± 1.6	9.5 ± 1.4	0.191
HDL (mmol/l)	1.3 ± 0.3	1.4 ± 0.4	0.069
LDL (mmol/l)	2.8 ± 0.4	2.9 ± 0.3	0.063
FBG (mmol/l)	6.3 ± 1.7	6.5 ± 2.0	0.485
SBP (mmHg)	137.2 ± 11.3	139.6 ± 12.1	0.183
DBP (mmHg)	82.5 ± 8.4	84.1 ± 8.6	0.221
NRG 1 (pg/ml)	264.8 ± 14.7	396.8 ± 19.9	<0.001
MoCA (points)	27.8 ± 1.1	20.9 ± 1.8	<0.001

Abbreviations: VaD: vascular dementia; HDL: high-density lipoprotein; LDL: low-density lipoprotein; FBG: fasting blood glucose; SBP: systolic blood pressure; DBP: diastolic blood pressure; NRG 1: neuregulin 1; MoCA: Montreal Cognitive Assessment.

**Table 2:** Correlation between MoCA and baseline characteristics in VaD.

	*r*	*p*
Age (years)	-0.323	0.284
Gender	-0.408	0.195
Education (years)	-0.667	0.317
HDL (mmol/l)	0.226	0.238
LDL (mmol/l)	-0.107	0.179
FBG (mmol/l)	-0.582	0.632
SBP (mmHg)	-0.465	0.503
DBP (mmHg)	-0.138	0.314
NRG 1 (pg/ml)	-0.374	0.036

Abbreviations: MoCA: Montreal Cognitive Assessment; VaD: vascular dementia; HDL: high-density lipoprotein; LDL: low-density lipoprotein; FBG: fasting blood glucose; SBP: systolic blood pressure; DBP: diastolic blood pressure; NRG 1: neuregulin 1.

**Table 3:** Multivariable analyses of MoCA and baseline characteristics in VaD.

	Regression coefficient	*p*	95% CI
Age (years)	0.199	0.132	0.118-1.007
Gender	0.246	0.284	0.221-1.098
Education (years)	0.433	0.249	0.163-1.106
HDL (mmol/l)	0.524	0.306	0.232-1.154
LDL (mmol/l)	0.317	0.108	0.186-1.113
FBG (mmol/l)	0.292	0.267	0.201-1.189
SBP (mmHg)	0.235	0.310	0.117-1.132
DBP (mmHg)	0.391	0.435	0.294-1.235
NRG 1 (pg/ml)	0.258	0.012	0.149-0.763

Abbreviations: MoCA: Montreal Cognitive Assessment; VaD: vascular dementia; HDL: high-density lipoprotein; LDL: low-density lipoprotein; FBG: fasting blood glucose; SBP: systolic blood pressure; DBP: diastolic blood pressure; NRG 1: neuregulin 1.

## Data Availability

The data used to support the findings of this study are available from the corresponding author upon request.

## References

[1] Li J., Li S., Song Y. (2020). Association of serum FAM19A5 with cognitive impairment in vascular dementia. *Disease Markers*.

[2] Wang Q., Yang W., Zhang J., Zhao Y., Xu Y. (2020). TREM2 overexpression attenuates cognitive deficits in experimental models of vascular dementia. *Neural Plasticity*.

[3] Xu Y., Wang Q., Qu Z., Yang J., Zhang X., Zhao Y. (2019). Protective effect of hyperbaric oxygen therapy on cognitive function in patients with vascular dementia. *Cell Transplantation*.

[4] Wang Q., Xu Y., Qi C., Liu A., Zhao Y. (2020). Association study of serum soluble TREM2 with vascular dementia in Chinese Han population. *The International Journal of Neuroscience*.

[5] Shao K., Shan S., Ru W., Ma C. (2020). Association between serum NPTX2 and cognitive function in patients with vascular dementia. *Brain and behavior*.

[6] Xu Y., Wang Q., Cui R., Lu K., Liu Y., Zhao Y. (2017). Uric acid is associated with vascular dementia in Chinese population. *Brain and behavior*.

[7] Xu Y., Wang Q., Liu Y., Cui R., Lu K., Zhao Y. (2016). Association between Helicobacter pylori infection and carotid atherosclerosis in patients with vascular dementia. *Journal of the Neurological Sciences*.

[8] Willem M. (2016). Proteolytic processing of neuregulin-1. *Brain Research Bulletin*.

[9] Hu X., Fan Q., Hou H., Yan R. (2016). Neurological dysfunctions associated with altered BACE1-dependent neuregulin-1 signaling. *Journal of Neurochemistry*.

[10] Ronchi G., Fornasari B. E., Crosio A. (2018). Chitosan tubes enriched with fresh skeletal muscle fibers for primary nerve repair. *BioMed Research International*.

[11] Fleck D., Voss M., Brankatschk B. (2016). Proteolytic processing of neuregulin 1 type III by three intramembrane-cleaving proteases. *The Journal of Biological Chemistry*.

[12] Iwakura Y., Wang R., Inamura N. (2017). Glutamate-dependent ectodomain shedding of neuregulin-1 type II precursors in rat forebrain neurons. *PLoS One*.

[13] Kataria H., Alizadeh A., Karimi-Abdolrezaee S. (2019). Neuregulin-1/ErbB network: an emerging modulator of nervous system injury and repair. *Progress in Neurobiology*.

[14] Román G. C., Tatemichi T. K., Erkinjuntti T. (1993). Vascular dementia: diagnostic criteria for research studies: report of the NINDS-AIREN International Workshop. *Neurology*.

[15] Gaebel W., Jessen F., Kanba S. (2018). Neurocognitive disorders in ICD-11: the debate and its outcome. *World Psychiatry*.

[16] Xu Y., Wang Q., Wu Z. (2019). The effect of lithium chloride on the attenuation of cognitive impairment in experimental hypoglycemic rats. *Brain Research Bulletin*.

[17] Zhang J., Tang L., Hu J., Wang Y., Xu Y. (2020). Uric acid is associated with cognitive impairment in the elderly patients receiving maintenance hemodialysis-a two-center study. *Brain and behavior*.

[18] Xu Y., Wang Q., Liu Y., Cui R., Zhao Y. (2016). Is helicobacter pylori infection a critical risk factor for vascular dementia?. *The International Journal of Neuroscience*.

[19] Peng Z., Jiang H., Wang X. (2019). The efficacy of cognitive training for elderly Chinese individuals with mild cognitive impairment. *BioMed Research International*.

[20] Shi L., Bergson C. M. (2020). Neuregulin 1: an intriguing therapeutic target for neurodevelopmental disorders. *Translational Psychiatry*.

[21] Xue W. K., Zhao W. J., Meng X. H., Shen H. F., Huang P. Z. (2019). Spinal cord injury induced neuregulin 1 signaling changes in mouse prefrontal cortex and hippocampus. *Brain Research Bulletin*.

[22] Xu J., Hu C., Chen S. (2017). Neuregulin-1 protects mouse cerebellum against oxidative stress and neuroinflammation. *Brain Research*.

[23] Simmons L. J., Surles-Zeigler M. C., Li Y., Ford G. D., Newman G. D., Ford B. D. (2016). Regulation of inflammatory responses by neuregulin-1 in brain ischemia and microglial cells in vitro involves the NF-kappa B pathway. *Journal of Neuroinflammation*.

[24] Navarro-González C., Huerga-Gómez A., Fazzari P. (2019). Nrg1 intracellular signaling is neuroprotective upon stroke. *Oxidative Medicine and Cellular Longevity*.

[25] Mei L., Xiong W. C. (2008). Neuregulin 1 in neural development, synaptic plasticity and schizophrenia. *Nature reviews Neuroscience*.

[26] Tian J., Geng F., Gao F. (2017). Down-regulation of neuregulin1/ErbB4 signaling in the hippocampus is critical for learning and memory. *Molecular Neurobiology*.

[27] Hei Y., Chen R., Mao X., Wang J., Long Q., Liu W. (2019). Neuregulin1 attenuates cognitive deficits and hippocampal CA1 neuronal apoptosis partly via ErbB4 receptor in a rat model of chronic cerebral hypoperfusion. *Behavioural Brain Research*.

[28] Jiang Q., Chen S., Hu C., Huang P., Shen H., Zhao W. (2016). Neuregulin-1 (Nrg1) signaling has a preventive role and is altered in the frontal cortex under the pathological conditions of Alzheimer’s disease. *Molecular Medicine Reports*.

[29] Xu J., de Winter F., Farrokhi C. (2016). Neuregulin 1 improves cognitive deficits and neuropathology in an Alzheimer’s disease model. *Scientific Reports*.

[30] Ryu J., Hong B. H., Kim Y. J. (2016). Neuregulin-1 attenuates cognitive function impairments in a transgenic mouse model of Alzheimer’s disease. *Cell Death & Disease*.

[31] Chang K. A., Shin K. Y., Nam E. (2016). Plasma soluble neuregulin-1 as a diagnostic biomarker for Alzheimer’s disease. *Neurochemistry International*.

[32] Mouton-Liger F., Dumurgier J., Cognat E. (2020). CSF levels of the BACE1 substrate NRG1 correlate with cognition in Alzheimer’s disease. *Alzheimer's Research & Therapy*.

